# Intelligibility and recall of sentences spoken by adult and child talkers wearing face masksa)

**DOI:** 10.1121/10.0006098

**Published:** 2021-09-08

**Authors:** Thanh Lan Truong, Andrea Weber

**Affiliations:** English Department, Eberhard Karls University, Wilhelmstrasse 50, 72074 Tübingen, Germany

## Abstract

With the Covid-19 pandemic, face masks have become part of our daily lives. While face masks are effective in slowing down the spread of the virus, they also make face-to-face communication more challenging. The present study sought to examine the impact of face masks on listeners' intelligibility and recall of sentences produced by one German native adult and one child talker. In the intelligibility task, German native adult listeners watched video clips of either an adult or a child talker producing sentences with and without a face mask. In a cued-recall experiment, another group of German native listeners watched the same video clips and then completed a cued-recall task. The results showed that face masks significantly affected listeners' intelligibility and recall performance, and this effect was equally true for both talkers. The findings here contribute to the fast growing and urgent research regarding the impact of face masks on communication.

## I. INTRODUCTION

With the Covid-19 pandemic, communication has changed. In particular, face masks present an additional challenge for listeners, as masks can modify the speech signal and at the same time conceal visual articulatory cues. Recent evidence suggests that, for adult talkers, both intelligibility and recall of what has been said can be negatively influenced by face masks (Smiljanic *et al.*, 2021; Truong *et al.*, 2021). But what about understanding and recalling what children have said? Children's voices are different from adult voices, and this could affect not only listeners' ability to understand and encode what has been said but also the relevance of visual articulatory information in face-to-face communication. The present study set out to investigate the intelligibility and recall of sentences spoken by a child talker in comparison to an adult talker when the talkers are wearing a face mask or not.

Visual cues, such as lip and jaw movements, can provide crucial linguistic information about speech sounds (e.g., Campbell, 2008; Summerfield, 1992). For example, lip closure is associated with a bilabial place of articulation as in the stop consonants /p/ and /b/, and the openness of the jaw correlates with the height of vowels (e.g., more open jaw for the vowel /a/ and less open jaw for /i/). Therefore, extracting information from a talker's visible articulators can supplement and complement information about speech sounds that is not included in the auditory signal (Sumby and Pollack, 1954). Indeed, information from both modalities is known to be automatically integrated during speech perception (e.g., Jesse and Massaro, 2010). Face masks, however, constrain access to visual articulatory information, and only auditory information is left for speech perception.

Face masks can potentially also degrade the acoustic signal itself by affecting the speech directivity and attenuating higher frequencies, which can result in a transmission loss and thus impact comprehension. While face coverings have been found to affect speech acoustics and comprehension in some studies (Corey *et al.*, 2020; Goldin *et al.*, 2020, Pörschmann *et al.*, 2020), other studies have found no such effects (e.g., Llamas *et al.*, 2009).

Generally speaking, any acoustically degraded speech (e.g., background noise or variation in pronunciations) provides listeners with less information, e.g., talkers varying considerably in their exact acoustic realization of phonemes and words as well as poor acoustic background conditions. For example, face masks can degrade the speech signal and have been found to affect the intelligibility of speech produced by adult talkers (e.g., Ernestus *et al.*, 2002; Peelle, 2018; Witteman *et al.*, 2014). These adverse listening conditions can thus make perceptual processing more effortful.

Given that listeners' cognitive resources are limited, mental resources in adverse listening conditions will be reallocated from memory back to perception (Pichora-Fuller, 2016). That is, additional effort can attenuate the listening challenges, but it may come at the expense of cognitive resources that might otherwise be available for memory encoding (see McCoy *et al.*, 2005; Rabbitt, 1968; Rönnberg *et al.*, 2013). Hence, degraded speech not only causes listeners to be less accurate in word identification, but at the same time it can negatively affect higher-level cognitive processing downstream, such as memory encoding (Pichora-Fuller, 2016; Rabbitt, 1968). Worse performance in recognizing previously heard words and recalling them has been found before in noisy conditions for conversational speaking styles and for unfamiliar accents (Gilbert *et al.*, 2014; Grohe and Weber, 2018; Keerstock and Smiljanic, 2019).

However, all previous intelligibility studies on face masks used audio recordings for their investigations, thus not considering how the lack of visual input when seeing a talker with a mask influences speech perception. Recently, Smiljanic *et al.* (2021) investigated this issue by using video recordings of two talkers (native vs non-native) producing two speaking styles (clear speech vs conversational speech) of a cohesive text. The video recordings were presented either in quiet or in the presence of different levels of competing speech noise. Their findings suggest that face masks did not negatively affect intelligibility of conversational native speech when no or little noise was present, but a negative effect emerged for higher noise levels. In comparison, for non-native speech, this negative mask-effect emerged already when little noise was present.

Concerning the effect of face masks on memory, Truong *et al.* (2021) previously found for German that face masks negatively affected memory encoding when sentences of an adult native talker were presented in quiet. That is, listeners recalled fewer words when the talker had been wearing a face mask than when the talker had not been wearing one. Smiljanic *et al.* (2021) also tested the impact of face masks on subsequent recall in English. While they found no mask-effect in quiet listening conditions for an adult native talker, an impact of face masks on memory was observed when sentences were mixed with noise. The difference in findings between the two studies could well be due to methodological differences that include the type of material (dissociated sentences vs cohesive text) and memory questions (only recall questions vs various question types). We expand on the findings of Truong *et al.* (2021) by investigating whether or not child speech produced with a face mask shows similar effects as adult speech.

Previous work on intelligibility and memory has almost exclusively investigated adult listeners' ability to understand and recall other adult talkers [but see Cooper *et al.* (2020)]. Here, we investigated word recognition and recall of sentences produced by a child talker in comparison to an adult talker. Children's voices can differ from adult voices on several acoustic and linguistic dimensions, which may impact listeners' ability to understand and encode what has been said by a child. More specifically, children's acoustic and linguistic properties differ from those of native adult speech (Lee *et al.*, 1999) such that children's speech is generally characterized by greater acoustic-phonetic variation and by overall higher fundamental frequency (F0) than native adult speech (Smith *et al.*, 1983; Tingley and Allen, 1975). For instance, a comparison of vowel productions by children and adults found that formant frequency averages of children are about 16% higher than those of adults [Kreiman and Sidtis (2013); but see Hillenbrand *et al.* (1995) and Vorperian and Kent (2007)]. This difference in F0 is largely due to distinct anatomical characteristics of children, who have a smaller larynx and shorter vocal folds. An eight-year-old child's vocal folds are, for example, about 8 mm long, while adult vocal folds are about 12–21 mm long. Because of these distinct physiological features, adult and child voices differ notably in F0 (Kreiman and Sidtis, 2013). At birth, an infant's F0 is at approximately 500 Hz, but by the time the child turns eight, F0 can be as low as 275 Hz, with little difference between boys and girls (Vorperian and Kent, 2007). While F0 remains relatively stable throughout the rest of childhood, children's speech motor control progressively increases until the age of 8–12 years, such that children gain better control over speaking rate, loudness, phonation, and pitch range, gradually meeting adult speech norms (Kent, 1976).

The child talker in the present study was nine years old. Even though pronunciation norms can already approach adult performance when children are five or six years old [i.e., there are only few segmental deviations or mispronunciations; see Vance *et al.* (2005)], children's voices are still higher than adult voices at that age (Vorperian and Kent, 2007). Since children have a higher F0 compared to adults, it is possible that the child speech produced with a mask is more affected by the mask and generally less intelligible than that of adult talkers, as face masks particularly attenuate higher frequencies. Additionally, limiting access to visual articulatory information through a face mask could further hamper performance, especially for child talkers.

The current paper investigated how face masks influence intelligibility (experiment A) and recall (experiment B) of spoken sentences. For the intelligibility experiment, we predicted for the adult and child talkers that a lack of visual cues would affect speech intelligibility negatively, such that recognition is less accurate when the sentences were produced with a face mask than without. This outcome would be in line with Smiljanic *et al.* (2021), who found that intelligibility was negatively affected by a face mask when native adult conversational speech was presented with a negative signal-to-noise ratio (SNR). Additionally, it was deemed possible that the child talker would be less intelligible overall and/or the negative effect of the mask could be enlarged for the child talker.

For the cued-recall experiment, the same recordings were used, but this time sentences were grouped in blocks and presented in quiet (see Truong *et al.*, 2021). We predicted similar findings to Truong *et al.* (2021), who used the same sentences and presented a subset of participants responding to the adult talker. Based on Truong *et al.* findings, we predicted that recall rates would be lower for sentences produced with a face mask compared to sentences produced without a face mask for both talkers. Such a result would be in congruence with the effortful hypothesis arguing that listeners must allocate additional cognitive resources when the listening situation is difficult, and this compromises subsequent memory encoding (Peelle, 2018). Additionally, if the child talker was harder to understand than the adult talker in the intelligibility task in experiment A or if not wearing a mask is particularly important for recall when the talker is a child, then the size of the mask-effect in experiment B might be larger for the child talker than for the adult talker.

## II. EXPERIMENT A

### A. Methods

#### 1. Participants

Eighty native German listeners between the ages of 18 and 36 years participated in the study [mean: 22.3, standard deviation (SD) = 3.2; 66 females] for a chance to take part in a monetary lottery. All participants reported that German was their first and dominant language. Participants were gathered through social media and university email. None of them reported hearing or vision impairments. Half of the participants watched an experimental version in which all sentences were produced by the female adult talker, and the other half watched a version in which all sentences were produced by the female child talker.

#### 2. Stimuli

**FIG. 1.**
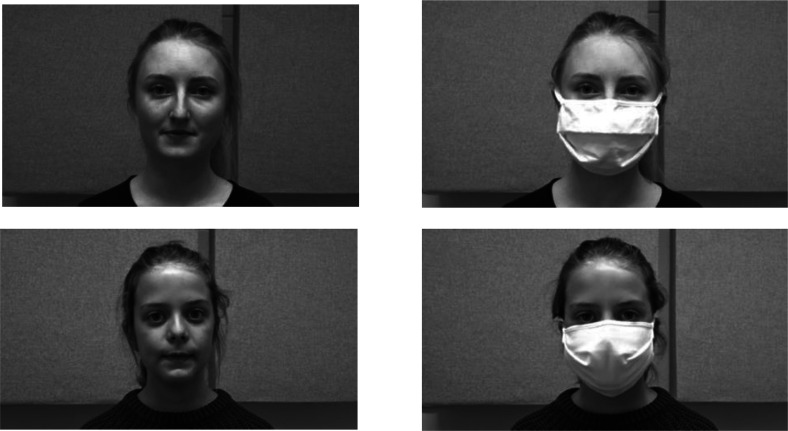
Representative screen shots for video recordings of both adult and child talker with and without a face mask. Videos were presented in color in the experiment.

The stimuli consisted of 48 meaningful but not highly predictable sentences, which were modelled after the Oldenburger Satztest (2000). Low predictability had the advantage that listeners could not easily guess individual words correctly without having understood them, since sentence context did not semantically constrain lexical options. The risk of a facilitatory influence of context was therefore relatively low and ensured a more thorough processing of the input (see, e.g., Rommers and Federmeier, 2018). The syntactic structure of all sentences was as follows: The sentences started with a determiner and a noun, followed by a verb, an adverb, an adjective, and a noun (e.g., *Die Köchin hilft montags armen Kindern*, “the cook helps on Mondays poor children”). Each content word occurred only once in the stimuli. The talkers selected for the experiment were a 22-year-old female adult native talker of German and a nine-year-old female child native talker of German, who both grew up in the south of Germany, where the experiment was also conducted (Fig. 1). The talkers were video recorded separately and produced all sentences with and without a face mask. The face mask consisted of two fabric layers: The inner layer was made of a thin fleece, and the outer layer was cotton. The talkers were instructed to produce all sentences at a normal speaking rate without hesitations or pauses and to not speak more clearly or loudly when wearing the mask. Recordings were made in a sound-attenuated room at the *LingTüLab* of Tübingen University. The talkers repeated the sentences until they were produced without any errors or hesitations. The videos were recorded by using a Sony (Tokyo, Japan) DSC-Hx90 camera with video resolution parameters set to FULL HD 1920 × 1080, capturing the head and shoulder of the talker. Audio was recorded at a sampling rate of 48 kHz with a high-quality microphone placed in front of the talker. Video recordings were segmented using iMovie. Audio was then detached from each segmented video and mixed with noise with a −12 dB SNR in Praat. As in the Bent and Bradlow (2003) intelligibility study, we used white noise, and the SNR level was chosen based on informal pre-testing that yielded an intermediate level of word identification rates for our sentences. While this level of noise can be considered profound, it was deemed necessary to stay clear from a ceiling performance for the chosen short sentences and words with high lexical frequency. The mixed audio clips were then reattached to the corresponding videos. The average F0 value of the adult talker was 235.5 Hz, and that of the child talker was 288.7 Hz (*t* = −39.35, *p* < 0.01). Durations for sentences produced by the adult talker without a mask were on average 3255 ms, and with a mask they were 3178 ms (*t* = −1.35 *p* = 0.18). Sentences produced by the child talker without a mask were on average 3997 ms long, and with a mask they were 3928 ms long (*t* = −1.13, *p* = 0.26). While spectral analysis [root mean square (rms) power] of the adult talker revealed no difference between sentences with (56.6 dB) and without a face mask (56.7 dB) (*t* = −0.28, *p* = 0.77), rms power for the child talker revealed a small but significant difference between sentences with (60.31 dB) and without a face mask (61.27 dB) (*t* = −4.2, *p* < 0.001).

#### 3. Procedure

The experiment was administered with the online software *SurveyGizmoLLC*, now called *Alchemer*. Participants were asked to wear headphones and participated online. We had emphasized in the instructions to use headphones and take part in the experiment on a computer, laptop, or tablet. Participants furthermore indicated after the experiment the type of device and headphones they had used. Prior to the experiment, they electronically signed written informed consent and were informed that they would listen to sentences mixed with noise. The experiment started with a practice trial and continued with the 48 experimental sentences recorded either with or without a face mask. During the practice trial, participants were asked to adjust the volume level to a comfortable listening level at which they could understand the sentences best and to keep it the same for the entire experiment. Mask condition was counterbalanced, and sentence order was randomized once, with half of the participants watching the video clips in the reverse order. After each video clip, a prompt with empty boxes for the words appeared on the screen and participants were asked to type in the sentence they had just heard. They were asked to type as many words as they had understood and to leave the box empty if they had not understood a word. The whole experiment lasted approximately 20–30 min. After the experimental session was completed, participants filled out a brief language background and were asked about technical problems, of which none were reported.

### B. Results

**FIG. 2.**
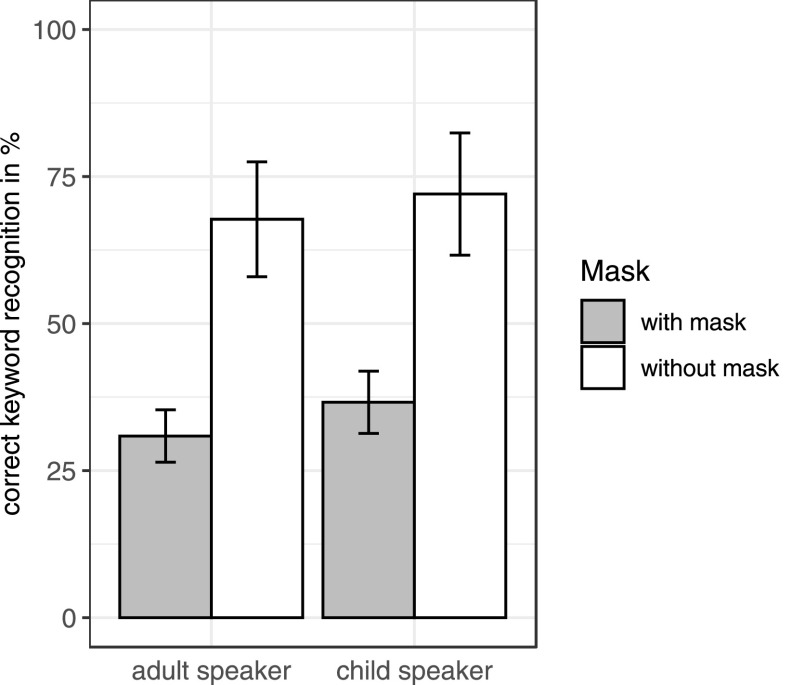
Average intelligibility scores for the adult and child talker in the conditions with and without face mask. The vertical bars represent standard errors.

For the purpose of the analysis, the initial determiner and noun (e.g., *die Köchin*, “the cook”) of the sentences were considered as one keyword, resulting in a total of five keywords for each sentence.^1^ The maximum number of correct keywords for each participant was therefore 240 (5 keywords × 48 sentences). Each keyword was scored as either correct (1) or incorrect (0) (Fig. 2). Scoring was done by T.L.T. and a research assistant. For any remaining uncertainties, A.W. was consulted. Overall, 51.8% of the keywords were identified as correct and 48.2% as incorrect.^2^ Correct identification responses included identical matches with the intended word forms (97.8% of the correct responses for the adult talker and 96.6% for the child talker) and what we categorized as typing errors (e.g., nonwords with mixed up letter order like *orndet* for *ordnet*, “orders,” and word forms with a minimal segmental difference that can be used in free variation in German like *gern/gerne*, “gladly”) (1.2% of the correct responses for the adult talker and 1.8% for the child talker). To assess the effect of face masks on listeners' keyword recognition accuracy, a logistic mixed-effects regression model (Jaeger, 2008) was incorporated using the lme4 package (Bates *et al.*, 2015) in R (version 4.0.5; R Core Team, 2021) with correct keyword recognition (success vs failure) as the dichotomous dependent variable. The model included face mask (mask vs no mask) and talker (adult vs child) as independent variables and face mask × talker as an interaction term. To account for additional variation, fixed factors of sentence duration and rms power were also included in the model. Items and participants were included as random crossed effects (Baayen *et al.*, 2008), with random intercepts and random slopes. The analysis showed a significant main effect of face mask [*b* = −1.70, standard error (SE) = 0.1, *p* < 0.001] and of rms power (*b* = 51.15, SE = 12.28, *p* < 0.001) on listeners' keyword recognition accuracy. Listeners recognized considerably fewer keywords accurately when the talkers had been wearing a mask (adult talker 31% correct; child talker 37% correct) compared to when the talkers had not been wearing a mask (adult talker 68% correct; child talker 72% correct). No main effect of talker was found (*b* = −0.05, SE = 0.15, *p* = 0.7), indicating that word recognition was comparable for both talkers. There was also no significant interaction between face mask and talker (*b* = −0.16, SE = 0.11, *p* = 0.14). The results thus suggest that intelligibility was considerably hampered when talkers were wearing a face mask, and this was equally true for the adult and child talker. This leaves open the question of whether the face masks produced an additional listening effort that came at the expense of memory encoding. To test for this possibility, we tested a new group of adult listeners using a cued-recall paradigm in experiment B.

## III. EXPERIMENT B

### A. Methods

#### 1. Participants

Eighty native German listeners between the ages of 19 and 56 years participated in the study (mean: 23.6; SD = 5; 63 females) for a chance to take part in a lottery.^3^ All participants reported that German was their first and dominant language. Participants were recruited through social media and university email. Two participants had to be excluded from further analyses since they did not follow the instructions. None of the participants reported hearing or vision impairments, and none had participated in experiment A. As in experiment A, half of the participants watched the videos produced by the adult talker, and the other half watched the videos produced by the child talker.

#### 2. Stimuli

Sentence recordings were identical to experiment A, but this time, the sentences were presented without noise. The 48 experimental sentences were divided into eight blocks of six sentences each. Sentence order was randomized once, and half of the participants watched the videos in the reverse order. The presence of a face mask was blocked, and blocks alternated between the mask and no-mask condition. The order of mask condition was counterbalanced, and sentences were presented with an interstimulus interval (ISI) of 2500 ms.

#### 3. Procedure

As in experiment A, participants took the online experiment on *Alchemer*. Participants were instructed to wear headphones and take part in the experiment on a computer, laptop, or tablet. Furthermore, participants indicated after the experiment the type of device and headphones they had used. Before the experiment started, participants digitally signed written informed consent. The experiment began with two practice trials during which participants could adjust the volume. After the practice trials, participants were asked not to change the volume for the 48 experimental sentences. The self-paced cued-recall task followed immediately after a block. For this task, sentences were presented up to the adverb orthographically on the screen (e.g., *Die Köchin hilft montags*, “the cook helps on Mondays”), and participants were asked to type in the missing two final words (e.g., *armen Kindern*, “poor children”) on their keyboard. Block length and cue length were determined based on informal pre-tests that yielded an intermediate level of recall rates. The recall cues were shown after each video block, on the order of block presentation, and participants could fill in their responses in any order. Then participants could press a button to initiate the next video block of six sentences. The whole experiment lasted approximately 20–30 min. After the experiment, participants filled out a short language background and were asked about technical problems, of which none were reported.

### B. Results

**FIG. 3.**
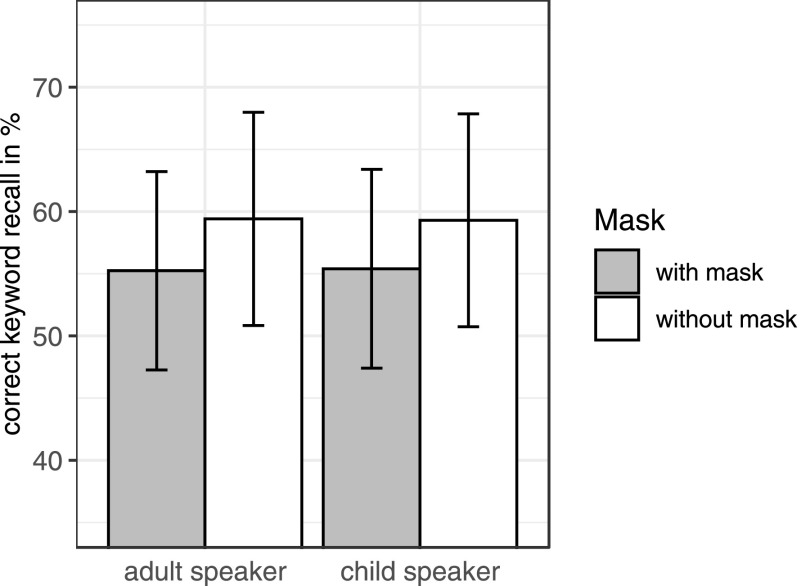
Average keyword recall scores for the adult and child talker in condition with and without face mask. The vertical bar represents standard errors.

Scoring was again done by T.L.T. and a research assistant. For any remaining uncertainties, A.W. was consulted. Each correctly recalled word received the score correct (1), while incorrectly recalled words or unrecalled words received the score incorrect (0) (Fig. 3). There were two keywords for each of the 48 sentences, making a total of 96 keywords to be recalled per participant. Overall, 57.3% of the words were recalled correctly and 42.7% incorrectly. Descriptive analyses of the incorrectly recalled words showed that most incorrectly recalled words had been complete omissions of a keyword (68% for the adult talker and 73% for the child talker). The remaining incorrect responses consisted of a variety of error types. Some responses were unrelated in form and in meaning to the intended words (e.g., *schwarze Schuhe*, “black shoes,” for *staubige Kissen*, “dusty pillows”), fewer responses were closely semantically related (e.g., *Ringe*, “rings,” for *Kreise*, “circles”), and a small number of responses consisted of phonetic errors involving a single sound change, like substitution, insertion, or deletion (e.g., *Schweine*, “pigs,” for *Steine*, “stones”) or typos (e.g., the nonword *Lmpen* for *Lampen*, “lamps”). Next, a logistic mixed-effects regression model with the lme4 package in R was employed to assess the effect of face masks on listeners' correctly recalled keywords (Bates *et al.*, 2015). Keyword recall (success vs failure) was the dichotomous dependent variable, and talker age (adult vs child), face mask (mask vs no mask), and block (8 blocks) were the independent variables; face mask × talker × block was added as an interaction term. To test linear and quadratic effects of block, orthogonal polynomials were used (Mirman, 2017). The same fixed effects and random intercepts were included as in experiment A. The analysis showed a significant effect of face mask, with listeners recalling fewer words when the talkers were wearing a mask (adult talker 55.2%; child talker 55.4%) compared to when the talkers were not wearing a mask (adult talker 59.4%; child talker 59.3%) (*b* = −0.21, SE = 0.08, *p* = 0.0003). There was a marginal effect for talker (*b* = −0.54, SE = 0.28, *p* = 0.05), indicating a trend for better recall rate for the child talker than for the adult talker. Recall performance was better for shorter sentence durations than for longer ones as the main effect for sentence duration showed (*b* = −0.36, SE = 0.15, *p* = 0.01). Further, a main effect for rms (*b* = −37.7, SE = 17.4, *p* = 0.03) was found, indicating that sentence recordings with less rms power were recalled better than sentences with higher rms power. There was no significant effect for block, and there were no interactions (all *p*-levels < 0.1). This result suggests that processing was easier when visual articulatory cues were available than when they were not present, and this availability left more cognitive resources for successful memory encoding. Overall, participants' recall performance was not worse for the child talker than for the adult talker. It thus appears that listening to the child's voice did not negatively affect recall.

## IV. DISCUSSION

The current study expanded on the results of Truong *et al.* (2021), who found that face masks can significantly impede the recall of sentences spoken by a native adult. To broaden the scope of Truong *et al.* findings, we investigated the impact of face masks on speech intelligibility and memory for adult and child speech. Adult listeners watched video clips of either an adult or a child talker producing sentences with and without a face mask embedded in noise (experiment A, intelligibility task) or in quiet (experiment B, cued-recall task). For both the intelligibility task and the recall task, it was found that performance was worse when the talkers had been wearing a face mask than when there was no mask.

Interestingly, the response patterns to the child talker did not differ substantially from the responses to the adult talker. The child talker in the current study was nine years old and contrasted in her average F0 from the adult speaker by 53.2 Hz. It was easy to notice that she was a child, and there were various reasons why talker age could have affected the results and impeded responses to the child talker. First, it was possible that white noise may have masked the high frequencies of the child talker more effectively than those of the adult talker. Second, children at that age can still vary more in their pronunciation than adults, and their formant values are higher across the board (Hillenbrand *et al.*, 1995). It is also conceivable that listeners previously experienced children as talkers who regularly deviate from canonical pronunciations and this experience could have been taken into consideration during comprehension of children's speech (see, e.g., Truong *et al.*, 2019). However, neither recognition rates in experiment A nor recall rates in experiment B were lower for the child talker than for the adult talker. Likewise, the size of the mask-effect did not differ for the two talkers, even though listeners may depend more heavily on visual speech cues in difficult listening situations (Xie *et al.*, 2014), and concealing these cues with a face mask could have been particularly detrimental for the child talker.

The only difference between the two talkers was marginally better recall rates for the child talker than the adult talker in experiment B. This difference could be related to the overall longer sentence durations of the child talker, which possibly enhanced memory encoding. However, the present data clearly indicate that in terms of a mask-effect, intelligibility and recall responses did not differ for the two talkers. There was thus never a disadvantage for the child talker. Since this is the first study we know of on the intelligibility and memory for child talkers, we do not know what would happen with younger talkers. The current talker was nine years old, and even though her pronunciation probably still varied more than an adult's pronunciation, there were no clear mispronunciations as one would find for younger children. Also, younger children have even higher average F0s. It is thus still possible that differences in intelligibility and recall, as well as a modulation of the mask-effect, would emerge for talkers younger than nine or in different communicative settings.

This brings us to the question of why face masks impeded recognition and recall in the current study. Face masks can change the acoustic signal but also hide visual articulatory information. While the current study did not set out to tease apart these two possible sources for mask-effects, we can still speculate on the primary reason for the observed impediment on recognition and recall. Previous work has shown that face masks have the potential to change the acoustic signal; observed changes range from negligible to substantial and depend on both the mask material and microphone position (e.g., Bottalico *et al.*, 2020; Corey *et al.*, 2020; Magee *et al.*, 2020). In the current study, spectral analyses showed no difference in rms values between the mask and no-mask condition for the adult talker and a small (<1 dB) but significant difference for the child talker. Despite the rms differences for the child talker, responses to the child talker were seemingly not less accurate. Had the acoustic signal itself been strongly affected by the mask, it should have also been harder to understand the audio recordings in quiet. We therefore presented in a *post hoc* test an additional 12 participants with the audio recordings in quiet and asked them to type the final two keywords after each sentence. Word recognition rates were overall very high and did not differ between the mask (adult 97.9% correct keywords; child 99.3% correct keywords) and no-mask conditions (adult 97.4% correct keywords; child 98.6% correct keywords). This ceiling effect, in combination with the small differences in rms, makes it unlikely that the signal itself was changed dramatically by the mask and that the missing visual cues due to the face masks were potentially the primary reason for the decrease in performance.

While our findings of experiment A are in line with the intelligibility results of Smiljanic *et al.* (2021), results of experiment B are at first glance in contrast with their results. In our recall experiment, for which the sentences were presented in quiet, we found a negative impact of face masks for both the adult talker [in line with Truong *et al.* (2021)] and the child talker. Smiljanic *et al.*, however, found a negative mask-effect for an adult talker only when noise was added with a negative SNR, not when sentences were presented in quiet. There are, however, several methodological differences across the two studies that could well explain the difference in findings. The two most important ones are arguably the employed materials and type of memory task. While the current study used dissociated sentences with low predictability, to avoid facilitatory influences of context, Smiljanic *et al.* used sentences from a coherent text, which made the listening environment more naturalistic. Encoding of cohesive information is, however, easier than encoding of dissociated information (Black and Bern, 1981), and this alleviation through cohesion possibly prevented a mask-effect when listening in quiet in Smiljanic *et al.* Also, in their memory task, Smiljanic *et al.* included different question types, ranging from fill in the blank and true/false to closed questions. These questions might well tap into different memory processes and could explain the difference in findings with the present study, which only tested recall memory with a fill in the blank task.

In summary, this study examined the effect of face masks on intelligibility and recall produced by adult and child talkers, and it makes an important contribution to the field's current understanding of the impact of face masks on speech perception. First, we found that face masks impede intelligibility and recall for both adult and child talkers equally. This finding should have implications in various communication situations, such as in classrooms, where information has to be understood and retained. Second, we established that intelligibility and recall were not worse for the child talker, certainly highlighting an encouraging observation that masked child speech is not disadvantaged more than adult speech in face-to-face communication. To our knowledge, the present work provided the first investigation of intelligibility and recall of sentences produced by adult and child talkers wearing face masks, laying a solid foundation for future research examining how face masks influence speech understanding for various talker and listener groups. Wearing a face mask is an essential means to slow the spread of Covid-19, and to further advance our understanding of the potential impact of face masks on communication is one step toward a better understanding of the impact of the pandemic.

## Footnotes

^1^
This was done because it was considered unlikely that participants would make a gender error between the determiner and noun. And indeed, none had made this mistake in the data.

^2^
Of all incorrectly identified keywords, the majority had been omissions, i.e., blank keyword boxes (70.0% for the adult talker; 77.3% for the child talker).

^3^
A subset of 32 participants listening to the adult speaker had been reported in Truong *et al.* (2021).
